# Supplementary material for: A high-resolution mRNA expression time course of embryonic development in zebrafish
Source: eLife. 2017 Nov 16;6:e30860. doi: 10.7554/eLife.30860 (PMC5690287; doi:10.7554/eLife.30860)
Supplement: Supplementary file 6. [file elife-30860-supp6.zip › biolayout-clusters-files/Cluster162-genes.html]

Cluster162


# Cluster162: Genes

| Ensembl ID | Gene Name | Chr | Start | End | Biotype |
| --- | --- | --- | --- | --- | --- |
| ENSDARG00000063474 | arfgef1 | 24 | 18592497 | 18732699 | protein\_coding |
| ENSDARG00000045506 | gramd4b | 25 | 19772652 | 19838647 | protein\_coding |
| ENSDARG00000086551 | irs4a | 21 | 43520497 | 43532022 | protein\_coding |
| ENSDARG00000078778 | lrch1 | 9 | 25285631 | 25359225 | protein\_coding |
| ENSDARG00000015860 | mtmr9 | 20 | 19045474 | 19084186 | protein\_coding |
| ENSDARG00000025902 | plekhg5a | 11 | 24227676 | 24301236 | protein\_coding |
| ENSDARG00000031200 | ppp2r5cb | 20 | 54108747 | 54174069 | protein\_coding |
| ENSDARG00000101982 | spryd3 | 23 | 36369852 | 36469853 | protein\_coding |
| ENSDARG00000044362 | unc119b | 21 | 39076395 | 39132506 | protein\_coding |
